# Performance comparison between multi-center histopathology datasets of a weakly-supervised deep learning model for pancreatic ductal adenocarcinoma detection

**DOI:** 10.1186/s40644-023-00586-3

**Published:** 2023-06-26

**Authors:** Francisco Carrillo-Perez, Francisco M. Ortuno, Alejandro Börjesson, Ignacio Rojas, Luis Javier Herrera

**Affiliations:** 1 Department of Computer Engineering, Automation and Robotics, University of Granada, Granada, Spain; 2 Clinical Bioinformatics Area, Fundación Progreso y Salud (FPS), Hospital Virgen del Rocío, Sevilla, Spain

**Keywords:** Digital pathology, Deep learning, Weakly-supervised classification

## Abstract

**Background:**

Pancreatic ductal carcinoma patients have a really poor prognosis given its difficult early detection and the lack of early symptoms. Digital pathology is routinely used by pathologists to diagnose the disease. However, visually inspecting the tissue is a time-consuming task, which slows down the diagnostic procedure. With the advances occurred in the area of artificial intelligence, specifically with deep learning models, and the growing availability of public histology data, clinical decision support systems are being created. However, the generalization capabilities of these systems are not always tested, nor the integration of publicly available datasets for pancreatic ductal carcinoma detection (PDAC).

**Methods:**

In this work, we explored the performace of two weakly-supervised deep learning models using the two more widely available datasets with pancreatic ductal carcinoma histology images, The Cancer Genome Atlas Project (TCGA) and the Clinical Proteomic Tumor Analysis Consortium (CPTAC). In order to have sufficient training data, the TCGA dataset was integrated with the Genotype-Tissue Expression (GTEx) project dataset, which contains healthy pancreatic samples.

**Results:**

We showed how the model trained on CPTAC generalizes better than the one trained on the integrated dataset, obtaining an inter-dataset accuracy of 90*.*62% ± 2*.*32 and an outer-dataset accuracy of 92*.*17% when evaluated on TCGA + GTEx. Furthermore, we tested the performance on another dataset formed by tissue micro-arrays, obtaining an accuracy of 98*.*59%. We showed how the features learned in an integrated dataset do not differentiate between the classes, but between the datasets, noticing that a stronger normalization might be needed when creating clinical decision support systems with datasets obtained from different sources. To mitigate this effect, we proposed to train on the three available datasets, improving the detection performance and generalization capabilities of a model trained only on TCGA + GTEx and achieving a similar performance to the model trained only on CPTAC.

**Conclusions:**

The integration of datasets where both classes are present can mitigate the batch effect present when integrating datasets, improving the classification performance, and accurately detecting PDAC across different datasets.

**Supplementary Information:**

The online version contains supplementary material available at 10.1186/s40644-023-00586-3.

## Introduction

Pancreatic ductal adenocarcinoma (PDAC) is a highly aggressive cancer type with a poor prognosis, with a rate below 10% of 5-year survival. However, early symptoms are almost non-existent, which makes early screening and detection difficult [1, 2]. The incidence of pancreatic cancer is growing year by year [3], and the majority of the patients (between 80%-85%) are found with locally advanced or distant metastatic disease, which highly reduces the prognosis [4]. Therefore, performing an accurate and early diagnosis of the disease is crucial to improve patient prognosis [5, 6].

Digital pathology is routinely used to diagnose patients, specifically, whole-slideimaging (WSI) using hematoxylin & eosin (H&E) stained tissue, where pathologists visually examine the tissue to find clinical histological patterns. However, this is a time-consuming task and does not allow the screening of multiple patients at the same time. With the recent advances in deep learning techniques in computer vision [7–9] and the increasing availability of publicly available datasets, the interest in creating clinical decision support systems (CDSS) using them is growing [10–12]. CDSS falls in the area of precision medicine, where machine learning and data analysis techniques are used on patient biological data to gain insights and provide accurate and personalized treatment.

In recent years, multiple models have been proposed in literature for PDAC identification. Fu et al. [13] presented a deep learning model for the classification of PDAC and control trained with a not publicly available dataset, obtaining good results (100% at WSI level). Similarly, Kronberg et al. [14] used transfer learning to classify between 5 classes (including control and PDAC), obtaining an accuracy of 95% in their test set. The training was performed in data obtained from tissue micro-arrays (TMAs). Li et al. [15] proposed the combination of histopathological and collagen fiber features using a graph neural network for the classification of control, chronic pancreatitis, and PDAC patients from TMAs, obtaining a final accuracy of 91*.*3%. Other image modalities have also been used for the detection of PDAC, such as Computer Tomography (CT) [16, 17] or Magnetic Resonance Imaging (MRI) [18, 19]. However, these works usually are applied over single databases, not comparing the performance obtained when trained from different sources, and how this might affect the performance.

Even though the number of available WSI in publicly accessible datasets is growing, the number is still low, limiting the potential of the trained models and their generalization capabilities. Two major datasets can be accessed containing images from PDAC patients, The Cancer Genome Atlas (TCGA) [20] and Clinical Proteomic Tumor Analysis Consortium (CPTAC) [21]. However, in the case of TCGA, not enough samples are available from the control class, limiting the creation of CDSS that distinguish between the classes. Other publicly available datasets can be leveraged, like the Genotype-Tissue Expression (GTEx) project [22], which contains multiple samples of healthy patients. By combining TCGA with GTEx, enough samples are available to train a deep learning model and thus obtain performance and generalization conclusions from an integrated dataset. However, the performance of deep learning models trained on integrated datasets for PDAC detection has not been yet explored, nor compared with the performance of models trained on a single dataset. Studies can be found in literature where it has been shown that the bias intrinsically present in digital pathology is arduous to omit, and that deep learning models usually pick these features over more characteristics ones [23]. Whether this issue is still persistent when data normalization is performed across different databases for pancreatic cancer detection has not been yet explored either, nor how it affect the classification performance.

In this work, we want to explore the limit performance of weakly-supervised deep learning models trained on two of the most used datasets in cancer research, TCGA, and CPTAC. Firstly, independent models will be trained on TCGA combined with GTEx and CPTAC respectively, to show their inter-dataset performance. Then, the pretrained models will be applied to the other one to show outer-dataset performance, and finally, to a TMAs dataset [15] to validate the obtained results.

## Materials and methods

### Data acquisition

Data for training the models were obtained from TCGA project, the GTEx project, and CPTAC. TCGA and GTEx datasets are combined in a single dataset, named as TCGA + GTEx for simplicity. PDAC and control samples were obtained from Li et al. work for validation purposes [15], which we named the tissue micro-arrays (TMA) dataset for simplicity purposes. For a more detailed description of this last dataset, we refer to the Materials and Methods section presented in the authors manuscript. Table 1 presents the number of samples per class and dataset. Patient IDs used from TCGA, GTEx and CPTAC are provided in Supplementary Material Tables 1, 2, 3 and 4.

**Table 1:** Number of samples available per class and per dataset used. One patient can have more than one sample

	TCGA	GTEx	TCGA + GTEx	CPTAC	TMA
Control	6	855	861	175	213
PDAC	203	0	203	382	380
Total	209	855	1064	557	593

### WSI preprocessing

Scanned WSIs, stained with hematoxylin and eosin, were acquired in SVS format and downsampled to 20 × magnification (0*.*5* µm* px^−1^). Typical WSIs easily supersede 10* k* × 10* k* pixels, and can therefore not be directly used as input in deep learning models. Instead, a regular grid was placed over the WSI resulting in smaller, nonoverlapping tiles of 256 × 256 pixels, consistent with related work in state-of-the-art WSI processing [24–26]. Slides were read using the OpenSlide Python package [27], which allows to efficiently deal with images in SVS format. The Otsu threshold method was used to obtain a mask of the tissue [28]. Tiles containing more than 60% background and with low-contrast were discarded. Up to 4000 tiles were obtained per slide. Then, tiles were saved as NumPy arrays in an HDF5 database per slice, for a faster reading and saving up space in comparison to saving tiles in other image formats.

To reduce staining biases that can be found when using data from multiple sources, stain normalization was used over the tiles during model training. To do so, we used the Slideflow Python library [29], which provides implementations of various normalization techniques. The Reinhard algorithm was used [30], specifically a fast version of it where the brightness standardization step was removed. We used the preset fit provided by the library to normalize our tiles.

### Weakly-supervised deep learning model and training details

TCGA + GTEx and CPTAC datasets were used to train two different classification models, while the TMA dataset was only used for validation purposes. A multiinstance learning (MIL) classification methodology was used for training the weaklysupervised WSI classifier. In a MIL methodology, a number *N* of tiles is grouped in what is usually called a bag of tiles. A bag is formed by *N* tiles, whose features are obtained using a CNN (taking the representation prior to the classification layers) and finally having *N* feature vectors from a particular dimension *D*. Once these feature vectors have been obtained, they can be fused in different ways. Multiple approaches have been proposed in the literature for digital pathology, from an average pooling of the features [31, 32] to using attention-based pooling [25, 26]. While attention-based pooling has proven to be a successful approach in recent works, we decided to use an average given the simplicity of the proposed task, expecting that using attention-based pooling would not improve results in our case but it will increase the model complexity and training time. Then, once the features have been fused, that final representation is forwarded through linear layers to obtain a prediction. The architecture is trained end-to-end, where both the classification layers and the feature extraction layers are trained at the same time. The model is trained in a weakly-supervised way given that we are assigning a global label for all tiles, even though not all of them might belong to that class (e.g. not all tiles in a tumor tissue might contain tumor cells).

**Fig. 1:**
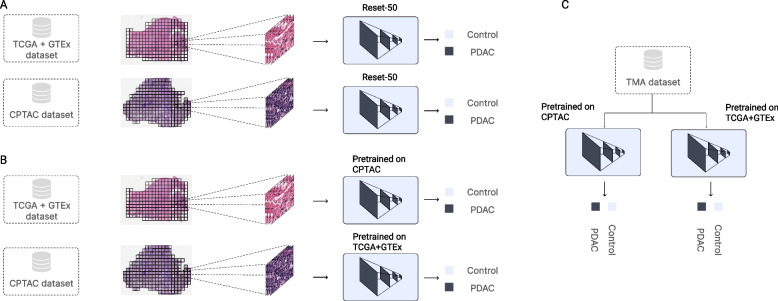
Multi-instance learning (MIL) pipeline used in this work and experiments performed. Panel **A** Independent classifiers are trained with a MIL classifier, using the CPTAC and TCGA + GTEx datasets independently. The classifier is trained using a bag of tiles (100 tiles) and a Resnet-50 pretrained on Imagenet for feature extraction. Then, the features are averaged-pool and forwarded through a linear layer to perform the final prediction. Panel **B** Once the models have been pretrained on each dataset respectively, their performance is evaluated on the other dataset, testing the generalization capabilities of each model. Panel **C** The pretrained models on CPTAC and TCGA + GTEx are tested in the TMA dataset respectively, to compare their performance

A patient-wise stratified 10-Fold CV was used to validate the methodology for both TCGA + GTEx and CPTAC (see Fig. 1A). This means that a given patient can only belong to one of the splits, being training, validation, or test. By doing so, we remove any possibility of having information leakage, and obtain more robust results. The training set was further split in 80–20% between training and validation. Then, both TCGA + GTEx and CPTAc datasets were fully used to train two independent models (using 20% of the dataset as validation), and then tested on the other one (TCGA + GTEx in the case of the CPTAC model, and CPTAC in the case of the TCGA + GTEx model) (see Fig. 1B). Finally, both pretrained models were tested on the TMA dataset (see Fig. 1C).

In this work, we have used a bag size of 100 tiles, and taken 200 random tiles per WSI, having a total of 2 bags per WSI. As the feature extractor network, we used a Resnet-50 pretrained on Imagenet, freezing all network weights except the last convolutional layer [33]. The final classification layer was formed by 2048 neurons, and its weights were initialized using the Xavier initialization method [34]. Data augmentation was used during training, applying vertical and horizontal flips. Also, tiles were normalized using the Imagenet mean and standard deviation, as usually performed when fine-tuning a pretrained network. The AdamW optimizer was used [35], with a learning rate of 1*e*^−3^, and a batch size of 64. In all cases, models were trained for 100 epochs using early-stopping criteria on the validation set performance with patience of 20 epochs. These hyperparameters were selected according to the results obtained in the validation sets of the 10-Fold CV.

Given that a given patient can potentially have more than one slide, to not obtain misleading results, we computed all metrics at the patient-level. To do so, we first obtained the output probabilities for all slices. Then, if a patient had multiple slices for the same class (tumor or control), we averaged the probabilities obtained per class and used the softmax function so they sum up to one. Then, the class with the maximum probability was the predicted class for a given patient. Thus, the final probability for a patient would be obtained as so:

1 $${{p}_{pat,c}}_{i}=\frac{{\sum }_{n=1}^{N}{p}_{{pat,c}_{i}}^{n}}{N}$$

where *N* is the number of slices available for patient *pat*, and *p*^*n*^_*pat,ci*_ is the probability obtained for the class *c*_*i*_ by the classification model for the slice *n*. Then, the softmax function is applied to the array of final probabilities.

Since patients can have slides from different classes (one from tumor tissue and one from solid tissue normal) we considered those as independent entities, given that they represent different classes. However, we obtained the patient-level prediction when, for a given patient and a given class, more than one slide was available. When only a single slide was available, the prediction made was used as the patient-level prediction. In the case of the TMA dataset, we followed the same approach given the identifiers provided by the authors. Since tissues from the same patient block are given the same label, we considered all of them for the classification at patient-level.

Models were trained using the Pytorch python package [36] and using one NVIDIA GeForce RTX 3060. The code and case IDs used in this work are available in the following Github repository^1^.

## Results

### Independent performance of TCGA + GTEX and CPTAC models

**Fig. 2:**
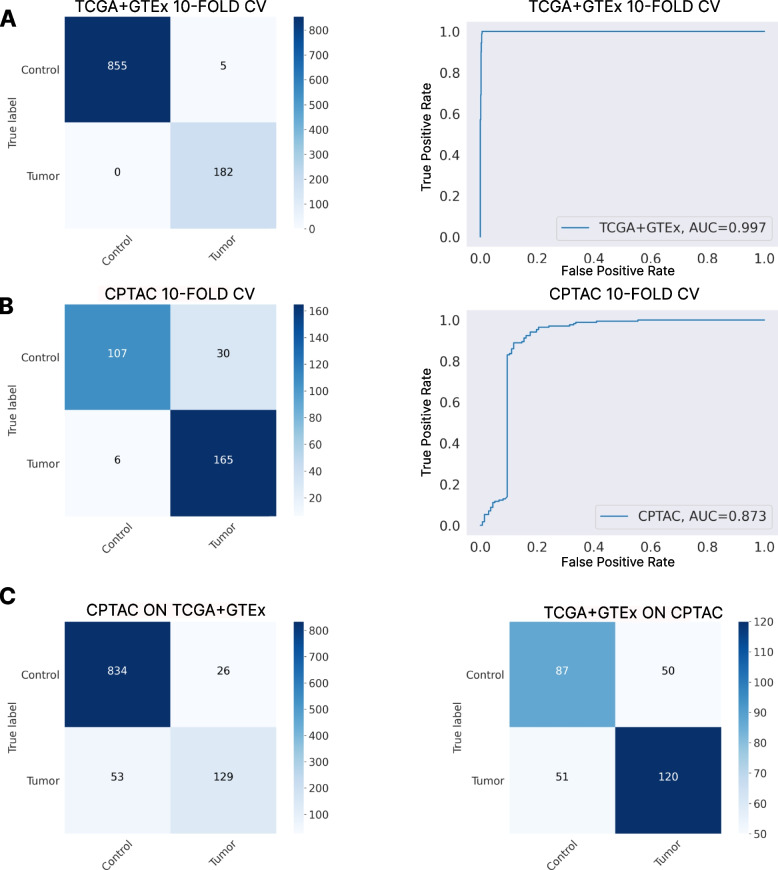
Classification performance of the independent models and their generalization. Panel **A** Confusion matrix and ROC curve obtained in the patient-wise stratified 10-Fold CV performed on the TCGA + GTEx data. Panel **B** Confusion matrix and ROC curve obtained in the patient-wise stratified 10-Fold CV performed on the CPTAC data. Panel **C** Confusion matrices obtained from the pretrained models, trained on CPTAC and TCGA + GTEx respectively, over the opposite dataset (CPTAC model predicting TCGA + GTEx data and TCGA + GTEx model predicting CPTAC data)

The classification performance of each independent model was evaluated with a patient-wise stratified 10-Fold CV. Metrics were obtained across the test sets on each split, and they were obtained at the patient-level. The TCGA + GTEx model obtained an accuracy of 99*.*0*.*4% ± 0*.*47, an F1-Score of 99*.*53% ± 0*.*47, and an AUC of 0*.*997 ± 0*.*003. Figure 2A shows the confusion matrix and ROC Curve obtained across all the dataset, by combining the results for every test set in the 10-Fold CV. On the other hand, the CPTAC model obtained an accuracy of 90*.*62% ± 2*.*32, an F1-Score of 88*.*07% ± 2*.*37, and an AUC of 0*.*872 ± 0*.*027. Figure 2B shows the confusion matrix and ROC Curve obtained across all the dataset, by combining the results for every test set in the 10-Fold CV. Only a few tumor patients are classified as control, which is desirable.

Then, we trained each model with the selected hyperparameters on all the data available in each dataset and tested their generalization performance on the other dataset, as well as on the TMA dataset. The pretrained model on CPTAC obtained an accuracy of 92*.*41%, an F1-Score of 92*.*17%, and an AUC of 0*.*839 when tested on TCGA + GTEx. The pretrained model on TGCA + GTEx obtained an accuracy of 67*.*20%, an F1-Score of 67*.*21%, and an AUC of 0*.*668 when tested on CPTAC. In Fig. 2C, the confusion matrices obtained for each model across all the test sets is presented. The difference between inter-dataset and outer-dataset performance is lower when the model has been trained using the CPTAC dataset with a 7*.*12% difference in terms of F1-Score, in comparison to the pretrained model on TCGA + GTEx tested on CPTAC, where the difference is 20*.*86% between the pretrained model and the one trained on CPTAC only. Therefore, if a better outerdataset performance wants to be achieved, the CPTAC dataset appears to be preferable.

**Fig. 3:**
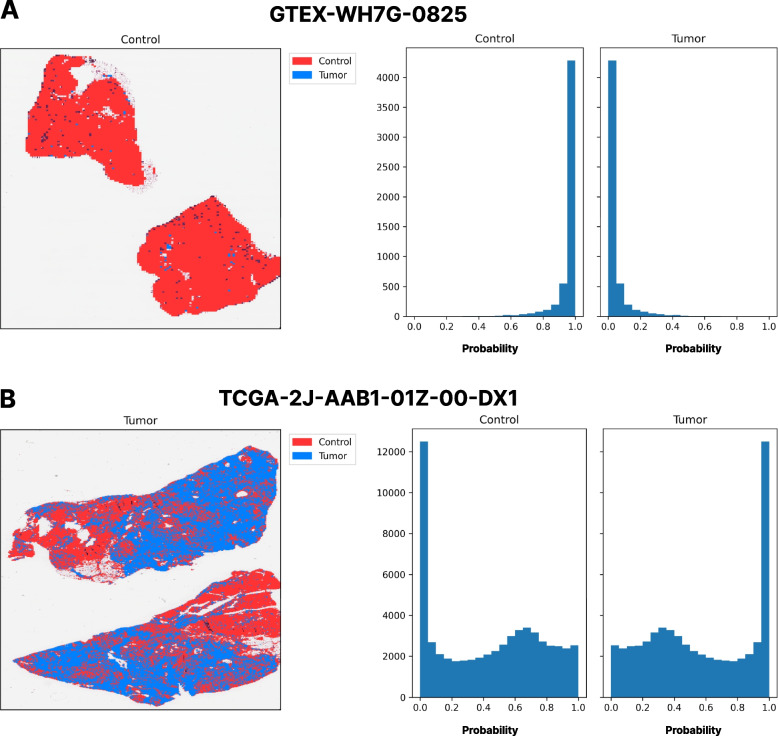
Prediction visualization across two samples by the CPTAC pretrained model. Panel **A** The majority of GTEx sample tissue tiles are predicted as a control, showing the discerning capabilities of the CPTAC pretrained model. Panel **B** The majority of the TCGA sample tissue tiles are predicted as Tumor. However, the TCGA samples also contain healthy tiles, that do not contain tumor cells. Thus, the CPTAC pretrained model also predicts some parts of the tissue as control

The predictions of the CPTAC models were obtained for two given samples in the TCGA + GTEx dataset, to visually inspect how they were distributed across the tissue (see Fig. 3). Figure 3A, shows the prediction distribution across the GTEx sample. The majority of the tiles are predicted as control with a high probability, as shown in the histogram presented. Figure 3B, shows the prediction distribution across the TCGA sample. Here we can observe how not all the tissue is predicted as tumor, but the majority of it is predicted as so. While the majority of the TCGA samples are formed by tumor tissue, normal tissue can also be found. This can be visualized by the CPTAC model predictions when the majority of the tiles are being predicted as tumor, but others are predicted as control. However, the final prediction would be assigned to the tumor class (see Fig. 3B histogram), which is the correct one.

### Generalization performance of TCGA + GTEX and CPTAC models

**Fig. 4:**
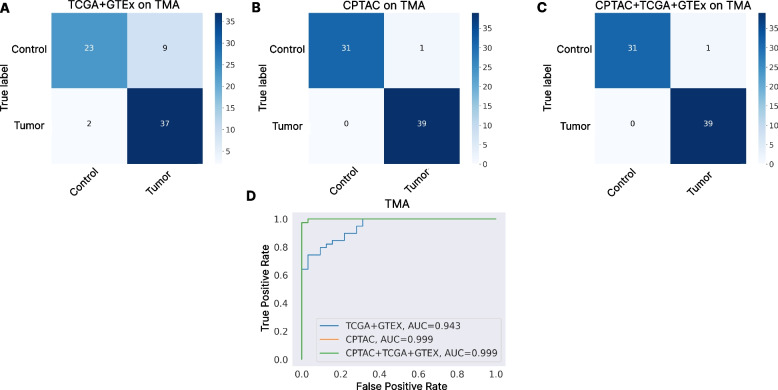
Generalization performance of CPTAC, TCGA + GTEx, and CPTAC + TCGA + GTEX pretrained models on the TMA dataset. Panel **A** Confusion matrix obtained by the pretrained model on TCGA + GTEx data on TMA. Panel **B** Confusion matrix obtained by the CPTAC pretrained model data on TMA. Panel **C** Confusion matrix obtained by the CPTAC + TCGA + GTEx pretrained model data on TMA. Panel **D** ROC Curves comparing CPTAC, TCGA + GTEx, and CPTAC + TCGA + GTEx pretrained models performance on the TMA dataset

The outer-dataset generalization capabilities of each model were tested on the TMA dataset. The CPTAC model obtained an accuracy of 98*.*59%, an F1-Score of 98*.*58%, and an AUC of 0*.*984. The TCGA + GTEx model obtained an accuracy of 84*.*50%, an F1-Score of 84*.*19%, and an AUC of 0*.*834. In Fig. 4A and B, the confusion matrices obtained with the TCGA + GTEx and the CPTAC models respectively are presented. There exists a considerable performance difference between the CPTAC and the TCGA + GTEx pretrained models, showing a greater generalization performance with the one pretrained on CPTAC. When comparing the ROC curves over the TMA dataset (see Fig. 4D), the CPTAC model outperforms the TCGA + GTEx model.

**Fig. 5:**
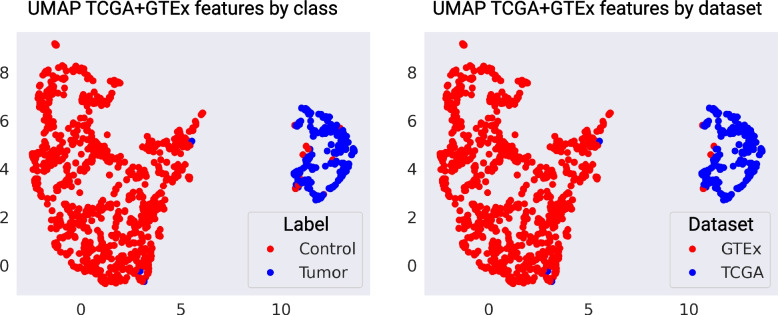
UMAP projection of features from TCGA + GTEx slides colored by label and dataset. Obtained features with the pretrained Resnet-50 on TCGA + GTEx are projected using the UMAP algorithm and visualized per label and dataset. The features are correctly separated between control and tumor. However, when the dataset plot is observed, control samples from TCGA are in the same cluster as the rest of the TCGA data, showing that the model is not accurately learning to differentiate between classes but between datasets

Given the low outer-dataset performance of the TCGA + GTEx model, we studied the features obtained by the deep learning model. When we plot the two-dimensional projection obtained with the UMAP algorithm, we can observe how the model is not learning to separate between the classes, but between the datasets, given that some control TCGA samples are plotted in the same region as the tumor TCGA samples (see Fig. 5 right). Even though the tiles stain is being normalized, it seems that the differences between the two datasets are greater than the difference between the classes, and that is shown when the model is applied to other datasets, where the performance highly decreases. The TCGA + GTEx model decreased performance on the TMA dataset confirms the results obtained on the CPTAC dataset, showing that this model has diminished generalization capabilities.

To test if this effect could be mitigated by including all the available data, we decided to perform three different experiments. First, a 10-Fold CV was performed over the TCGA + GTEx data, but all the CPTAC data was included on each training split. Then, the same experiment was performed but over the CPTAC data, adding TCGA + GTEx to the training splits. Finally, a single model was trained over all three databases, and tested on the TMA dataset. In the 10-Fold experiment were TCGA + GTEx data was added to the CPTAC training sets, an accuracy of 90*.*62 ± 5*.*44, an F1-score of 86*.*78 ± 5*.*44, and an AUC of 0*.*865 ± 0*.*05, improving the accuracy obtained by only training on CPTAC data, but increasing the standard deviation. In the 10-Fold experiment were CPTAC data was added to the TCGA + GTEx training sets, an accuracy of 98*.*09 ± 0*.*85, an F1-score of 99*.*06 ± 0*.*83, and an AUC of 0*.*987 ± 0*.*01, similarly to what is obtained when using TCGA + GTEx alone. When the model trained on CPTAC + TCGA + GTEx was tested on the TMA database, an accuracy of 99*.*04%, an F1-Score of 99*.*24%, and an AUC of 0*.*992 ± 1*.*39 were obtained, which is the same performance the model trained only on CPTAC obtained and it is better than using TCGA + GTEx alone, fixing the problems obtained with this integrated dataset. The confusion matrix obtained is presented in Fig. 4C, and the comparison of the ROC curves obtained per model can be observed in Fig. 4D.

**Fig. 6:**
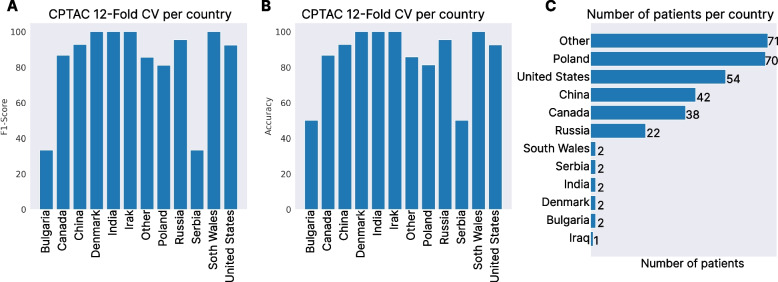
Classification performance in a 12-Fold CV overt the CPTAC dataset where each test set was formed by patients of a single nationality. Panel **A** Test accuracy obtained per country in the 12-Fold CV. Panel **B** F1-score obtained per country in the 12-Fold CV. Panel **C** Number of samples per nationality in the CPTAC dataset

To further test the generalization capabilities of the CPTAC-trained model, we decided to perform a 12-Fold CV where each test split would contain only patients from a given nationality, which were obtained from the original publication [37]. The accuracy, F1-Score, and the number of patients per country are presented in Fig. 6. The mean accuracy obtained was 92*.*58% ± 17*.*26 and the F1-score was 83*.*41% ± 23*.*19. The model was able to obtain a performance above 80% on 10 out of the 12 countries, where the two countries below the 80% only contained two samples, impacting how much a misclassified sample affects the performance. Also, the model was able to obtain a 100% accuracy in 4 out of the 12 countries. Given that the performance could be affected by those countries with a low number of samples, we also computed the accuracy and F1-Score of the six countries with a sufficient number of samples (Other, Poland, United States, China, Canada, and Russia). The accuracy obtained was 89*.*18% ± 4*.*84, and the F1-Score 89*.*05% ± 4*.*95.

## Discussion

Data homogenization is a growing concern in digital pathology. More datasets are being released, whose data have been obtained with different technologies and variations in the staining procedure, which affects the final visual characteristics. Deep learning models rely on these features for learning the downstream task, therefore, these variations affect their final performance. Works presented in literature have shown how deep learning models are prone to capture tissue biases when trained for diagnostic tasks, mitigating their real performance [23]. However, if these effects also affect the detection of PDAC had not been yet studied in literature.

To improve patient prognosis an early diagnosis of PDAC is crucial. The use of deep learning models can provide a fast first clinical opinion that can be then validated by the expert clinician. However, to create robust models sufficiently diverse and properly pre-processed datasets are necessary. Unfortunately, biomedical data is still scarce, and the necessity of integrating databases is still present. We have tested the major publicly available databases containing cancer samples, and that support the creation of deep learning models. We have confirmed the findings obtained by Howard et al. [23], by showing that when TCGA and GTEx are integrated, a weakly-supervised WSI deep learning model learned features that accurately separate the two datasets, but are not significant enough to properly distinguish between classes, and thus, having a good outer-dataset performance (see Figs. 2C and 4D). Since we are integrating datasets that almost solely contain samples from a given class, there could exist an implicit bias, that makes the model learn to separate the datasets and not the classes. However, integrating multiple datasets with diverse samples is something that is routinely done with gene expression data [38] after multiple preprocessing and integration steps (such as batch correction), and it was something that we wanted to test in the context of digital pathology.

This also confirms what authors presented in their work, that stain normalization might not be enough for reducing these tissue location biases [23]. This is something that does not happen when the model was trained only on the CPTAC data, showing that the harmonization of a dataset is crucial to have a better generalization performance, and needs to be thoughtfully considered [39]. Nevertheless, we showed how this effect can be mitigated when harmonized data is included in other databases. When the model was trained using all three databases, the performance equals to the one obtained by CPTAC, having the same outer-dataset performance. On the other hand, this effect did not happend when we included TCGA + GTEx data in the training during a 10-Fold evaluation over CPTAC. Thus, it seems that less diverse datasets, where there are not samples from all classes can benefit from integrating datasets that contain both classes, but including class-specific samples might decrease the final performance of a model. Therefore, for performing an accurate PDAC detection by training a deep learning model, CPTAC data by itself or the combination of all three databases should be used. To further validate the generalization capabilities of a weakly-supervised deep learning model, we performed a 12-Fold CV per patient country in CPTAC, showing how the model was able to accurately classify patients from different nationalities even when they are not part of the training dataset.

## Conclusions

In this work, we have shown how by using a dataset that has been harmonized, improved results can be obtained in terms of classification performance when compared with the integration of two different datasets. Even though stain normalization and value normalization have been applied to reduce color variability, the state-of-theart methodology for WSI classification has learned features that accurately separate the two datasets, but are not significant enough to properly distinguish between classes, and thus, having a good outer-dataset performance. The final proposed model, trained on CPTAC, is able to accurately distinguish between PDAC and control patients, in both inter and outer-dataset scenarios. Thus, a more fine-grained homogenization is required when training deep learning models, to have improved performance when facing new samples. To mitigate this effect, enough data needs to be used from both classes, to revert the lower performance. We demonstrated it when training a deep learning model over all three datasets, showing that the performance was increased over using only the integration of TCGA + GTEx. Thus, both using CPTAC alone or a combination of CPTAC + TCGA + GTEx, can serve as a solution of training weakly-supervised deep learning models and accurately detect PDAC on histology imaging.

In future work, we would like to explore the generalization capabilities of these models in a more complex problem, classifying control, PDAC, and pancreatitis.

This could show further show the importance of data homogenization.

### Supplementary Information

**Additional file 1: Table 1.** TCGA Patient IDs used in this work. **Table 2.** GTEx Patient IDs used in this work. **Table 3.** CPTAC Patient IDs used in this work. **Table 4.** TMA dataset Patient IDs used in this work

## Data Availability

Those Case IDs used in this work and the code to reproduce the experiments are available in the following URL: https://github.com/pacocp/WSI-Pancreas-Classification.

## Abbreviations

TCGA: The Cancer Genome Atlas
GTEx: Genotype-Tissue Expression
CPTAC: Clinical Proteomic Tumor Analysis Consortium
MIL: Multi-instance Learning
PDAC: Pancreatic Ductal Adenocarcinoma
TMAs: Tissue Micro-Arrays
WSI: Whole-Slide-Imaging

## Footnotes

1 https://github.com/pacocp/WSI-Pancreas-Classification
