# Correction: Performance comparison between multi-center histopathology datasets of a weakly-supervised deep learning model for pancreatic ductal adenocarcinoma detection

**DOI:** 10.1186/s40644-023-00636-w

**Published:** 2023-11-17

**Authors:** Francisco Carrillo-Perez, Francisco M. Ortuno, Alejandro Börjesson, Ignacio Rojas, Luis Javier Herrera

**Affiliations:** 1 Department of Computer Engineering, Automation and Robotics, University of Granada, Granada, Spain; 2 Clinical Bioinformatics Area, Fundación Progreso y Salud (FPS), Hospital Virgen del Rocío, Sevilla, Spain

Following publication of the original article [1], we have been notified of a change needed in the Funding section.

- Originally published Funding section:

This work was funded by the Spanish Ministry of Sciences, Innovation and Universities under Project PID2021-128317OB-I00 and the projects from Junta de Andalucia P20-00163.

- Corrected Funding section:

This work is part of grant PID2021-128317OB-I00 funded by MCIN/AEI/10.13039/501100011033 and of Project P20-00163 funded by Consejería de Universidad, Investigación e Innovación, both also funded by “ERDF A way of making Europe”.

The original article has been corrected.
