# Supplementary material for: Performance comparison between multi-center histopathology datasets of a weakly-supervised deep learning model for pancreatic ductal adenocarcinoma detection
Source: Cancer Imaging. 2023 Jun 26;23:66. doi: 10.1186/s40644-023-00586-3 (PMC10294485; doi:10.1186/s40644-023-00586-3)
Supplement: Supplementary file 1 — Additional file 1: Table 1. TCGA Patient IDs used in this work. Table 2. GTEx Patient IDs used in this work. Table 3. CPTAC Patient IDs used in this work. Table 4. TMA dataset Patient IDs used in this work [file 40644_2023_586_MOESM1_ESM.pdf]

RESEARCH

Performance comparison between multi-center histopathology datasets of a weakly-supervised deep learning model for pancreatic ductal adenocarcinoma detection

Francisco Carrillo-Perez*, Francisco M. Ortuno, Alejandro Börjesson, Ignacio Rojas and Luis Javier Herrera

*Correspondence:
franciscocp@ugr.es
Full list of author information is available at the end of the article

Table 1: TCGA Patient IDs used in this work

TCGA Patient IDs
TCGA-2J-AAB1, TCGA-2J-AAB4, TCGA-2J-AAB6, TCGA-2J-AAB8, TCGA-2J-AAB9, TCGA-2J-AABA, TCGA-2J-AABE, TCGA-2J-AABF, TCGA-2J-AABH, TCGA-2J-AABI, TCGA-2J-AABK, TCGA-2J-AABO, TCGA-2J-AABP, TCGA-2J-AABR, TCGA-2J-AABT, TCGA-2J-AABU, TCGA-2J-AABV, TCGA-2L-AAQA, TCGA-2L-AAQE, TCGA-2L-AAQI, TCGA-2L-AAQJ, TCGA-2L-AAQL, TCGA-2L-AAQM, TCGA-3A-A9I5, TCGA-3A-A9I7, TCGA-3A-A9I9, TCGA-3A-A9IB, TCGA-3A-A9IC, TCGA-3A-A9IH, TCGA-3A-A9IJ, TCGA-3A-A9IL, TCGA-3A-A9IN, TCGA-3A-A9IO, TCGA-3A-A9IR, TCGA-3A-A9IS, TCGA-3A-A9IU, TCGA-3A-A9IV, TCGA-3A-A9IX, TCGA-3A-A9IZ, TCGA-3A-A9J0, TCGA-3E-AAAY, TCGA-3E-AAAZ, TCGA-F2-6879, TCGA-F2-6880, TCGA-F2-7273, TCGA-F2-7276, TCGA-F2-A44G, TCGA-F2-A44H, TCGA-F2-A7TX, TCGA-F2-A8YN, TCGA-FB-A4P5, TCGA-FB-A4P6, TCGA-FB-A545, TCGA-FB-A5VM, TCGA-FB-A78T, TCGA-FB-A7DR, TCGA-FB-AAPP, TCGA-FB-AAPQ, TCGA-FB-AAPS, TCGA-FB-AAPU, TCGA-FB-AAPY, TCGA-FB-AAPZ, TCGA-FB-AAQ0, TCGA-FB-AAQ1, TCGA-FB-AAQ2, TCGA-FB-AAQ3, TCGA-FB-AAQ6, TCGA-FZ-5919, TCGA-FZ-5920, TCGA-FZ-5921, TCGA-FZ-5922, TCGA-FZ-5923, TCGA-FZ-5924, TCGA-FZ-5926, TCGA-H6-A45N, TCGA-H8-A6C1, TCGA-HV-A5A3, TCGA-HV-A5A4, TCGA-HV-A5A5, TCGA-HV-A5A6, TCGA-HV-A7OL, TCGA-HV-A7OP, TCGA-HV-AA8V, TCGA-HV-AA8X, TCGA-HZ-7289, TCGA-HZ-7918, TCGA-HZ-7919, TCGA-HZ-7920, TCGA-HZ-7922, TCGA-HZ-7923, TCGA-HZ-7924, TCGA-HZ-7925, TCGA-HZ-7926, TCGA-HZ-8001, TCGA-HZ-8002, TCGA-HZ-8003, TCGA-HZ-8005, TCGA-HZ-8315, TCGA-HZ-8317, TCGA-HZ-8519, TCGA-HZ-8636, TCGA-HZ-8637, TCGA-HZ-8638, TCGA-HZ-A49G,
Continued on next page

Table 1 – continued from previous page

**TCGA Patient IDs**
TCGA-HZ-A49H, TCGA-HZ-A49I, TCGA-HZ-A4BH, TCGA-HZ-A4BK, TCGA-HZ-A77O, TCGA-HZ-A77P, TCGA-HZ-A77Q, TCGA-HZ-A8P0, TCGA-HZ-A8P1, TCGA-HZ-A9TJ, TCGA-IB-7644, TCGA-IB-7645, TCGA-IB-7646, TCGA-IB-7647, TCGA-IB-7649, TCGA-IB-7651, TCGA-IB-7652, TCGA-IB-7654, TCGA-IB-7885, TCGA-IB-7886, TCGA-IB-7887, TCGA-IB-7888, TCGA-IB-7889, TCGA-IB-7890, TCGA-IB-7891, TCGA-IB-7893, TCGA-IB-7897, TCGA-IB-8126, TCGA-IB-8127, TCGA-IB-A5SO, TCGA-IB-A5SP, TCGA-IB-A5SQ, TCGA-IB-A5SS, TCGA-IB-A5ST, TCGA-IB-A6UF, TCGA-IB-A6UG, TCGA-IB-A7LX, TCGA-IB-A7M4, TCGA-IB-AAUM, TCGA-IB-AAUN, TCGA-IB-AAUO, TCGA-IB-AAUP, TCGA-IB-AAUQ, TCGA-IB-AAUR, TCGA-IB-AAUS, TCGA-IB-AAUT, TCGA-IB-AAUU, TCGA-IB-AAUV, TCGA-IB-AAUW, TCGA-L1-A7W4, TCGA-LB-A7SX, TCGA-LB-A8F3, TCGA-LB-A9Q5, TCGA-M8-A5N4, TCGA-OE-A75W, TCGA-PZ-A5RE, TCGA-Q3-A5QY, TCGA-Q3-AA2A, TCGA-RB-A7B8, TCGA-RB-AA9M, TCGA-RL-AAAS, TCGA-S4-A8RM, TCGA-S4-A8RO, TCGA-S4-A8RP, TCGA-US-A774, TCGA-US-A776, TCGA-US-A779, TCGA-US-A77E, TCGA-US-A77G, TCGA-US-A77J, TCGA-XD-AAUG, TCGA-XD-AAUH, TCGA-XD-AAUI, TCGA-XD-AAUL, TCGA-XN-A8T3, TCGA-XN-A8T5, TCGA-YB-A89D, TCGA-YY-A8LH, TCGA-Z5-AAPL

Table 2: GTEx Patient IDs used in this work

**GTEx Patient IDs**
GTEX-1117F, GTEX-111CU, GTEX-111FC, GTEX-111VG, GTEX-111YS, GTEX-1122O, GTEX-1128S, GTEX-113JC, GTEX-117XS, GTEX-117YW, GTEX-117YX, GTEX-1192W, GTEX-1192X, GTEX-11DXX, GTEX-11DXY, GTEX-11DXZ, GTEX-11DYG, GTEX-11DZ1, GTEX-11EI6, GTEX-11EMC, GTEX-11EQ8, GTEX-11EQ9, GTEX-11GS4, GTEX-11GSO, GTEX-11GSP, GTEX-11I78, GTEX-11ILO, GTEX-11LCK, GTEX-11NSD, GTEX-11NUK, GTEX-11O72, GTEX-11OF3, GTEX-11ONC, GTEX-11P7K, GTEX-11P81, GTEX-11PRG, GTEX-11TT1, GTEX-11TTK, GTEX-11TUW, GTEX-11UD2, GTEX-11VI4, GTEX-11WQC, GTEX-11WQK, GTEX-11XUK, GTEX-11ZTS, GTEX-11ZTT, GTEX-11ZU8, GTEX-11ZUS, GTEX-11ZVC, GTEX-1211K, GTEX-1212Z, GTEX-12584, GTEX-12696, GTEX-1269C, GTEX-12BJ1, GTEX-12KS4, GTEX-12WS9, GTEX-12WSA, GTEX-12WSB, GTEX-12WSC, GTEX-12WSD, GTEX-12WSE, GTEX-12WSF, GTEX-12WSG, GTEX-12WSH, GTEX-12WSI, GTEX-12WSK, GTEX-12WSL,

Continued on next page

Table 2 – continued from previous page

GTEx Patient IDs
GTEX-12WSM, GTEX-12WSN, GTEX-12ZZW, GTEX-12ZZX,
GTEX-12ZZY, GTEX-12ZZZ, GTEX-13111, GTEX-13112,
GTEX-1313W, GTEX-1314G, GTEX-131XE, GTEX-131XF,
GTEX-131XG, GTEX-131XH, GTEX-131XW, GTEX-131YS,
GTEX-132AR, GTEX-132NY, GTEX-132Q8, GTEX-132QS,
GTEX-1339X, GTEX-133LE, GTEX-1399Q, GTEX-1399R,
GTEX-1399S, GTEX-1399T, GTEX-1399U, GTEX-139D8,
GTEX-139TS, GTEX-139TT, GTEX-139TU, GTEX-139UC,
GTEX-139UW, GTEX-139YR, GTEX-13CF2, GTEX-13CF3,
GTEX-13CIG, GTEX-13CZV, GTEX-13D11, GTEX-13FH7,
GTEX-13FHO, GTEX-13FHP, GTEX-13FLV, GTEX-13FLW,
GTEX-13FTW, GTEX-13FTX, GTEX-13FTY, GTEX-13FXS,
GTEX-13G51, GTEX-13IVO, GTEX-13JUV, GTEX-13JVG,
GTEX-13N11, GTEX-13N1W, GTEX-13N2G, GTEX-13NYB,
GTEX-13NYC, GTEX-13NYS, GTEX-13NZ8, GTEX-13NZ9,
GTEX-13NZA, GTEX-13NZB, GTEX-13O1R, GTEX-13O21,
GTEX-13O3O, GTEX-13O3P, GTEX-13O3Q, GTEX-13O61,
GTEX-13OVG, GTEX-13OVH, GTEX-13OVI, GTEX-13OVJ,
GTEX-13OVL, GTEX-13OW5, GTEX-13OW6, GTEX-13OW7,
GTEX-13OW8, GTEX-13PDP, GTEX-13PL6, GTEX-13PL7,
GTEX-13PVQ, GTEX-13PVR, GTEX-13QIC, GTEX-13QJ3,
GTEX-13QJC, GTEX-13RTJ, GTEX-13S7M, GTEX-13S86,
GTEX-13SLW, GTEX-13SLX, GTEX-13U4I, GTEX-13VXT,
GTEX-13VXU, GTEX-13W3W, GTEX-13W46, GTEX-13X6H,
GTEX-13X6I, GTEX-13X6J, GTEX-13X6K, GTEX-13YAN,
GTEX-1445S, GTEX-144FL, GTEX-144GL, GTEX-144GM,
GTEX-144GN, GTEX-144GO, GTEX-145LS, GTEX-145LU,
GTEX-145LV, GTEX-145ME, GTEX-145MF, GTEX-145MG,
GTEX-145MH, GTEX-145MI, GTEX-145MN, GTEX-145MO,
GTEX-146FH, GTEX-146FQ, GTEX-146FR, GTEX-14753,
GTEX-1477Z, GTEX-147F3, GTEX-147F4, GTEX-147GR,
GTEX-147JS, GTEX-148VI, GTEX-148VJ, GTEX-1497J,
GTEX-14A5H, GTEX-14A5I, GTEX-14A6H, GTEX-14ABY,
GTEX-14AS3, GTEX-14ASI, GTEX-14BIL, GTEX-14BIM,
GTEX-14BIN, GTEX-14BMU, GTEX-14BMV, GTEX-14C38,
GTEX-14C39, GTEX-14C5O, GTEX-14DAQ, GTEX-14DAR,
GTEX-14E1K, GTEX-14E6C, GTEX-14E6D, GTEX-14E6E,
GTEX-14E7W, GTEX-14ICK, GTEX-14ICL, GTEX-14JFF,
GTEX-14JG1, GTEX-14JG6, GTEX-14JIY, GTEX-14LLW,
GTEX-14LZ3, GTEX-14PHW, GTEX-14PHX, GTEX-14PHY,
GTEX-14PII, GTEX-14PJ2, GTEX-14PJ3, GTEX-14PJ4,
GTEX-14PJ6, GTEX-14PJM, GTEX-14PJN, GTEX-14PJO,
GTEX-14PK6, GTEX-14PKU, GTEX-14PKV, GTEX-14PN3,

Continued on next page

Table 2 – continued from previous page

GTEx Patient IDs
GTEX-14PN4, GTEX-14PQA, GTEX-14XAO, GTEX-15CHC,
GTEX-15CHQ, GTEX-15CHR, GTEX-15CHS, GTEX-15D1Q,
GTEX-15D79, GTEX-15DCD, GTEX-15DCZ, GTEX-15DDE,
GTEX-15DYW, GTEX-15DZA, GTEX-15EO6, GTEX-15EOM,
GTEX-15ER7, GTEX-15ETS, GTEX-15EU6, GTEX-15FZZ,
GTEX-15G19, GTEX-15G1A, GTEX-15RIE, GTEX-15RIF,
GTEX-15RJ7, GTEX-15RJE, GTEX-15SB6, GTEX-15SDE,
GTEX-15SHU, GTEX-15SHV, GTEX-15SHW, GTEX-15SKB,
GTEX-15SZO, GTEX-15TU5, GTEX-15UF6, GTEX-15UF7,
GTEX-15UKP, GTEX-16A39, GTEX-16GPK, GTEX-16MT8,
GTEX-16MT9, GTEX-16MTA, GTEX-16NFA, GTEX-16NPV,
GTEX-16NPX, GTEX-16XZZ, GTEX-16YQH, GTEX-16Z82,
GTEX-178AV, GTEX-17EUY, GTEX-17EVP, GTEX-17EVQ,
GTEX-17F96, GTEX-17F97, GTEX-17F98, GTEX-17F9E,
GTEX-17F9Y, GTEX-17HGU, GTEX-17HHE, GTEX-17HHY,
GTEX-17HII, GTEX-17JCI, GTEX-17KNJ, GTEX-17MF6,
GTEX-183WM, GTEX-18464, GTEX-18465, GTEX-18A66,
GTEX-18A67, GTEX-18A6Q, GTEX-18A7A, GTEX-18A7B,
GTEX-18D9B, GTEX-18D9U, GTEX-19HZE, GTEX-1A32A,
GTEX-1A3MW, GTEX-1A3MX, GTEX-1A8FM, GTEX-1A8G6,
GTEX-1A8G7, GTEX-1AMFI, GTEX-1AX8Y, GTEX-1AX8Z,
GTEX-1AX9I, GTEX-1AX9J, GTEX-1AX9K, GTEX-1AYCT,
GTEX-1AYD5, GTEX-1B8KE, GTEX-1B8KZ, GTEX-1B8L1,
GTEX-1B8SF, GTEX-1B8SG, GTEX-1B932, GTEX-1B933,
GTEX-1B97I, GTEX-1B97J, GTEX-1B98T, GTEX-1B996,
GTEX-1BAJH, GTEX-1C2JI, GTEX-1C4CL, GTEX-1C64N,
GTEX-1C64O, GTEX-1C6VQ, GTEX-1C6VR, GTEX-1C6VS,
GTEX-1C6WA, GTEX-1CAMQ, GTEX-1CAMR, GTEX-1CAMS,
GTEX-1CAV2, GTEX-1CB4F, GTEX-1CB4G, GTEX-1CB4H,
GTEX-1CB4I, GTEX-1CB4J, GTEX-1E1VI, GTEX-1E2YA,
GTEX-1EH9U, GTEX-1EKGG, GTEX-1EMGI, GTEX-1EN7A,
GTEX-1EU9M, GTEX-1EWIQ, GTEX-1EX96, GTEX-1F48J,
GTEX-1F52S, GTEX-1F5PK, GTEX-1F5PL, GTEX-1F6I4,
GTEX-1F6IF, GTEX-1F6RS, GTEX-1F75A, GTEX-1F75B,
GTEX-1F75I, GTEX-1F75W, GTEX-1F88F, GTEX-1FIGZ,
GTEX-1GF9U, GTEX-1GF9V, GTEX-1GF9W, GTEX-1GF9X,
GTEX-1GL5R, GTEX-1GMR3, GTEX-1GMR8, GTEX-1GMRU,
GTEX-1GN1U, GTEX-1GN1V, GTEX-1GN1W, GTEX-1GN2E,
GTEX-1GN73, GTEX-1GPI6, GTEX-1GTWX, GTEX-1GZ2Q,
GTEX-1GZ4H, GTEX-1GZ4I, GTEX-1GZHY, GTEX-1H11D,
GTEX-1H1CY, GTEX-1H1DE, GTEX-1H1DF, GTEX-1H1DG,
GTEX-1H1E6, GTEX-1H1ZS, GTEX-1H23P, GTEX-1H2FU,
GTEX-1H3NZ, GTEX-1H3O1, GTEX-1H3VE, GTEX-1H3VY,
Continued on next page

Table 2 – continued from previous page

GTEx Patient IDs
GTEX-1H4P4, GTEX-1HB9E, GTEX-1HBPH, GTEX-1HBPI, GTEX-1HBPM, GTEX-1HC8U, GTEX-1HCU6, GTEX-1HCU7, GTEX-1HCU8, GTEX-1HCU9, GTEX-1HCVE, GTEX-1HFI6, GTEX-1HGF4, GTEX-1HKZK, GTEX-1HR98, GTEX-1HR9M, GTEX-1HSGN, GTEX-1HSKV, GTEX-1HSMO, GTEX-1HSMP, GTEX-1HSMQ, GTEX-1HT8W, GTEX-1HUB1, GTEX-1I19N, GTEX-1I1CD, GTEX-1I1GP, GTEX-1I1GQ, GTEX-1I1GR, GTEX-1I1GS, GTEX-1I1GT, GTEX-1I1GU, GTEX-1I1GV, GTEX-1I4MK, GTEX-1I6K6, GTEX-1ICG6, GTEX-1ICLY, GTEX-1ICLZ, GTEX-1IDFM, GTEX-1IDJC, GTEX-1IDJD, GTEX-1IDJE, GTEX-1IDJH, GTEX-1IDJI, GTEX-1IDJU, GTEX-1IDJV, GTEX-1IE54, GTEX-1IGQW, GTEX-1IKJJ, GTEX-1IKK5, GTEX-1IKOE, GTEX-1IKOH, GTEX-1IL2U, GTEX-1IL2V, GTEX-1IOXB, GTEX-1IY9M, GTEX-1J1OQ, GTEX-1J1R8, GTEX-1J8EW, GTEX-1J8JJ, GTEX-1J8Q2, GTEX-1J8Q3, GTEX-1JJ6O, GTEX-1JJE9, GTEX-1JJEA, GTEX-1JK1U, GTEX-1JKYN, GTEX-1JKYR, GTEX-1JMLX, GTEX-1JMOU, GTEX-1JMPZ, GTEX-1JMQI, GTEX-1JMQJ, GTEX-1JMQK, GTEX-1JMQL, GTEX-1JN1M, GTEX-1JN6P, GTEX-1K2DA, GTEX-1K2DU, GTEX-1K9T9, GTEX-1KAFJ, GTEX-1KANA, GTEX-1KANB, GTEX-1KD4Q, GTEX-1KWVE, GTEX-1KXAM, GTEX-1L5NE, GTEX-1LB8K, GTEX-1LC46, GTEX-1LG7Y, GTEX-1LG7Z, GTEX-1LGRB, GTEX-1LH75, GTEX-1LKK1, GTEX-1LSNL, GTEX-1LSNM, GTEX-1LSVX, GTEX-1LVA9, GTEX-1LVAN, GTEX-1LVAO, GTEX-1M4P7, GTEX-1M5QR, GTEX-1MA7X, GTEX-1MCC2, GTEX-1MCQQ, GTEX-1MCYP, GTEX-1MGNQ, GTEX-1MJIX, GTEX-1MJK2, GTEX-1MUQO, GTEX-1N2DV, GTEX-1N2DW, GTEX-1N2EE, GTEX-1N2EF, GTEX-1N5O9, GTEX-1N7R6, GTEX-1NSGN, GTEX-1NUQO, GTEX-1NV5F, GTEX-1NV8Z, GTEX-1O97I, GTEX-1O9I2, GTEX-1OFPY, GTEX-1OJC4, GTEX-1OKEX, GTEX-1OZHM, GTEX-1P4AB, GTEX-1PBJI, GTEX-1PBJJ, GTEX-1PDJ9, GTEX-1PFEY, GTEX-1PIIG, GTEX-1POEN, GTEX-1PPGY, GTEX-1PPH6, GTEX-1PPH7, GTEX-1PPH8, GTEX-1PWST, GTEX-1QAET, GTEX-1QCLZ, GTEX-1QEPI, GTEX-1QL29, GTEX-1QMI2, GTEX-1QP28, GTEX-1QP29, GTEX-1QP2A, GTEX-1QP66, GTEX-1QP67, GTEX-1QP6S, GTEX-1QPFJ, GTEX-1QW4Y, GTEX-1R46S, GTEX-1R7EU, GTEX-1R7EV, GTEX-1R9JW, GTEX-1R9K4, GTEX-1R9K5, GTEX-1R9PM, GTEX-1R9PN, GTEX-1R9PO, GTEX-1RAZA, GTEX-1RAZQ, GTEX-1RAZR, GTEX-1RAZS, GTEX-1RB15, GTEX-1RDX4, GTEX-1RNSC, GTEX-1RQED, GTEX-1S3DN, GTEX-1S5VW, GTEX-1S5ZU, GTEX-1S82P, GTEX-1S82Z,
Continued on next page

Table 2 – continued from previous page

GTEx Patient IDs
GTEX-1S831, GTEX-1S83E, GTEX-N7MS, GTEX-N7MT, GTEX-NFK9, GTEX-NL3G, GTEX-NL3H, GTEX-NL4W, GTEX-NPJ7, GTEX-NPJ8, GTEX-O5YT, GTEX-O5YU, GTEX-O5YV, GTEX-OHPJ, GTEX-OHPK, GTEX-OHPL, GTEX-OHPM, GTEX-OHPN, GTEX-OIZF, GTEX-OIZG, GTEX-OIZH, GTEX-OIZI, GTEX-OOBJ, GTEX-OOBK, GTEX-OXRK, GTEX-OXRL, GTEX-OXRN, GTEX-OXRO, GTEX-P44G, GTEX-P44H, GTEX-P4PP, GTEX-P4PQ, GTEX-P4QR, GTEX-P4QS, GTEX-P4QT, GTEX-P78B, GTEX-PLZ4, GTEX-PLZ5, GTEX-PLZ6, GTEX-POMQ, GTEX-POYW, GTEX-PSDG, GTEX-PVOW, GTEX-PW2O, GTEX-PWCY, GTEX-PWO3, GTEX-PWOO, GTEX-PX3G, GTEX-Q2AG, GTEX-Q2AH, GTEX-Q2AI, GTEX-Q734, GTEX-QCQG, GTEX-QDT8, GTEX-QDVJ, GTEX-QDVN, GTEX-QEG4, GTEX-QEG5, GTEX-QEL4, GTEX-QESD, GTEX-QLQ7, GTEX-QLQW, GTEX-QMR6, GTEX-QMRM, GTEX-QV31, GTEX-QV44, GTEX-QVJO, GTEX-QVUS, GTEX-QXCU, GTEX-R3RS, GTEX-R45C, GTEX-R53T, GTEX-R55C, GTEX-R55D, GTEX-R55E, GTEX-R55F, GTEX-R55G, GTEX-REY6, GTEX-RM2N, GTEX-RN5K, GTEX-RN64, GTEX-RNOR, GTEX-RTLS, GTEX-RU72, GTEX-RUSQ, GTEX-RVPU, GTEX-RVPV, GTEX-RWS6, GTEX-RWSA, GTEX-S32W, GTEX-S33H, GTEX-S3LF, GTEX-S3XE, GTEX-S4P3, GTEX-S4Z8, GTEX-S7PM, GTEX-S7SE, GTEX-S95S, GTEX-SE5C, GTEX-SIU7, GTEX-SIU8, GTEX-SJXC, GTEX-SN8G, GTEX-SNOS, GTEX-SUCS, GTEX-T2IS, GTEX-T2YK, GTEX-T5JC, GTEX-T5JW, GTEX-T6MN, GTEX-TKQ2, GTEX-TML8, GTEX-TMMY, GTEX-TSE9, GTEX-U3ZH, GTEX-U3ZM, GTEX-U3ZN, GTEX-U412, GTEX-U4B1, GTEX-U8T8, GTEX-U8XE, GTEX-UJHI, GTEX-UJMC, GTEX-UPIC, GTEX-UPJH, GTEX-UPK5, GTEX-UTHO, GTEX-V1D1, GTEX-V955, GTEX-VJWN, GTEX-VJYA, GTEX-VUSG, GTEX-W5WG, GTEX-W5X1, GTEX-WFG7, GTEX-WFG8, GTEX-WFJO, GTEX-WFON, GTEX-WH7G, GTEX-WHPG, GTEX-WHSB, GTEX-WHSE, GTEX-WHWD, GTEX-WI4N, GTEX-WK11, GTEX-WL46, GTEX-WOFL, GTEX-WQUQ, GTEX-WRHK, GTEX-WRHU, GTEX-WVJS, GTEX-WVLH, GTEX-WWTW, GTEX-WWYW, GTEX-WXYG, GTEX-WY7C, GTEX-WYBS, GTEX-WYJK, GTEX-WYVS, GTEX-WZTO, GTEX-X15G, GTEX-X261, GTEX-X3Y1, GTEX-X4EO, GTEX-X4EP, GTEX-X4LF, GTEX-X4XX, GTEX-X4XY, GTEX-X585, GTEX-X5EB, GTEX-X62O, GTEX-X8HC, GTEX-XBEC, GTEX-XBED, GTEX-XBEW, GTEX-XGQ4, GTEX-XK95, GTEX-XLM4, GTEX-XMD1, GTEX-XMD2, GTEX-XMD3, GTEX-XMK1, GTEX-XOT4, GTEX-XOTO, GTEX-XPVG, GTEX-XQ3S, GTEX-XQ8I, GTEX-XUJ4, GTEX-XUW1, GTEX-XV7Q, GTEX-XXEK, GTEX-XYKS, GTEX-Y111, GTEX-Y114, GTEX-Y3I4, GTEX-Y3IK, GTEX-Y5LM, GTEX-Y5V5, GTEX-Y5V6, GTEX-Y8E4, GTEX-Y8LW, GTEX-Y9LG, GTEX-YB5E, GTEX-YB5K, GTEX-YEC3, GTEX-YEC4, GTEX-YECK, GTEX-YF7O, GTEX-YFC4, GTEX-YFCO, GTEX-YJ89, GTEX-YJ8A, GTEX-YJ8O, GTEX-Z93S, GTEX-ZAB4,
Continued on next page

Table 2 – continued from previous page

GTEx Patient IDs
GTEX-ZAB5, GTEX-ZAJG, GTEX-ZAK1, GTEX-ZC5H, GTEX-ZDTS,
GTEX-ZDTT, GTEX-ZDYS, GTEX-ZE7O, GTEX-ZE9C, GTEX-ZEX8,
GTEX-ZF28, GTEX-ZF29, GTEX-ZF2S, GTEX-ZF3C, GTEX-ZG7Y,
GTEX-ZGAY, GTEX-ZLFU, GTEX-ZLV1, GTEX-ZLWG, GTEX-ZP4G,
GTEX-ZPCL, GTEX-ZPIC, GTEX-ZPU1, GTEX-ZQG8, GTEX-ZT9W,
GTEX-ZT9X, GTEX-ZTPG, GTEX-ZTTD, GTEX-ZU9S, GTEX-ZUA1,
GTEX-ZV68, GTEX-ZV6S, GTEX-ZV7C, GTEX-ZVE1, GTEX-ZVE2,
GTEX-ZVP2, GTEX-ZVT2, GTEX-ZVT3, GTEX-ZVT4, GTEX-ZVZP,
GTEX-ZVZQ, GTEX-ZWKS, GTEX-ZXES, GTEX-ZXG5, GTEX-ZY6K,
GTEX-ZYFC, GTEX-ZYFD, GTEX-ZYFG, GTEX-ZYT6, GTEX-ZYVF,
GTEX-ZYW4, GTEX-ZYWO, GTEX-ZYY3, GTEX-ZZPT, GTEX-ZZPU

Table 3: CPTAC Patient IDs used in this work

CPATC Patient IDs
C3L-00017, C3L-00102, C3L-00189, C3L-00277, C3L-00395,
C3L-00401, C3L-00589, C3L-00598, C3L-00599, C3L-00622,
C3L-00625, C3L-00640, C3L-00819, C3L-00881, C3L-00885,
C3L-00928, C3L-01031, C3L-01032, C3L-01036, C3L-01036,
C3L-01037, C3L-01037-, C3L-01051, C3L-01052, C3L-01053,
C3L-01054, C3L-01124, C3L-01158, C3L-01160, C3L-01328,
C3L-01453, C3L-01598, C3L-01637, C3L-01662, C3L-01687,
C3L-01689, C3L-01702, C3L-01703, C3L-01971, C3L-02109,
C3L-02112, C3L-02115, C3L-02116, C3L-02118, C3L-02463,
C3L-02604, C3L-02606, C3L-02610, C3L-02613, C3L-02701,
C3L-02809, C3L-02890, C3L-02897, C3L-02899, C3L-03123,
C3L-03126, C3L-03129, C3L-03350, C3L-03356, C3L-03356-,
C3L-03371, C3L-03388, C3L-03394, C3L-03395, C3L-03624,
C3L-03628, C3L-03630, C3L-03632, C3L-03635, C3L-03639,
C3L-03743, C3L-04027, C3L-04072, C3L-04080, C3L-04473,
C3L-04475, C3L-04479, C3L-04495, C3L-04848, C3L-04853,
C3N-00198, C3N-00249, C3N-00302, C3N-00303, C3N-00436,
C3N-00511, C3N-00512, C3N-00513, C3N-00514, C3N-00516,
C3N-00517, C3N-00518, C3N-00709, C3N-00954, C3N-00957,
C3N-01011, C3N-01012, C3N-01165, C3N-01166, C3N-01167,
C3N-01168, C3N-01169, C3N-01375, C3N-01378, C3N-01379,
C3N-01380, C3N-01381, C3N-01382, C3N-01383, C3N-01388,
C3N-01389, C3N-01502, C3N-01714, C3N-01715, C3N-01716,
C3N-01719, C3N-01897, C3N-01900, C3N-01907, C3N-01997,
C3N-01998, C3N-02010, C3N-02069, C3N-02295, C3N-02573,
C3N-02579, C3N-02585, C3N-02589, C3N-02590, C3N-02591,

Continued on next page

Table 3 – continued from previous page

**CPTAC Patient IDs**
C3N-02592, C3N-02697, C3N-02768, C3N-02939, C3N-02940, C3N-02944, C3N-02971, C3N-02997, C3N-02998, C3N-03000, C3N-03006, C3N-03007, C3N-03039, C3N-03061, C3N-03069, C3N-03086, C3N-03100, C3N-03173, C3N-03190, C3N-03211, C3N-03426, C3N-03428, C3N-03430, C3N-03439, C3N-03440, C3N-03665, C3N-03666, C3N-03670, C3N-03754, C3N-03778, C3N-03780, C3N-03839, C3N-03840, C3N-03853, C3N-03854, C3N-03884, C3N-04119, C3N-04126, C3N-04282, C3N-04283, C3N-04284

Table 4: TMA dataset Patient IDs used in this work

**TMA dataset Patient IDs**
BBS14011_C, BBS14011_D, BIC14011b_C, BIC14011b_D, BIC14011b_F, HPan-Ade170Sur_A, HPan-Ade170Sur_B, HPan-Ade170Sur_C, HPan-Ade170Sur_D, HPan-Ade170Sur_E, HPan-Ade170Sur_F, HPan-Ade170Sur_G, HPan-Ade170Sur_H, HPan-Ade170Sur_I, HPan-Ade170Sur_J, HPan-Ade170Sur_K, HPan-Ade180Sur_A, HPan-Ade180Sur_B, HPan-Ade180Sur_C, HPan-Ade180Sur_D, HPan-Ade180Sur_E, HPan-Ade180Sur_F, HPan-Ade180Sur_G, HPan-Ade180Sur_H, HPan-Ade180Sur_I, HPan-Ade180Sur_J, HPan-Ade180Sur_K, HPan-Qde120Sur_A, HPan-Qde120Sur_B, HPan-Qde120Sur_C, HPan-Qde120Sur_D, HPan-Qde120Sur_E, HPan-Qde120Sur_F, HPan-Qde120Sur_G, HPan-Qde120Sur_H, HPan-Qde120Sur_I, PA485_F, PA802_A, PA802_B, PA802_C, PA802_D, PA802_E, PA802_F
